# Effectiveness of a thrombin-gelatin flowable for treating severe liver bleeding: an experimental study

**DOI:** 10.1186/s12876-023-03114-6

**Published:** 2024-02-14

**Authors:** Francisco J Sánchez-del-Valle, María-Isabel Sánchez-Seco, Adrián González Jiménez, Florentina Acosta, Pedro Fernández-Domínguez, Juan-José Pérez-Alegre

**Affiliations:** 1 General and Digestive Unit, Central Hospital of Defense Gómez Ulla, Glorieta del Ejército, 1, Madrid, (28047) Spain; 2 University of Alcalá de Henares, Madrid, Spain; 3 Complutense University, Madrid, Spain; 4 Experimental Surgery Unit, Central Hospital of Defense Gómez Ulla, Madrid, Spain

**Keywords:** Hemostatic, Thrombin, Swine, Liver surgery, Experimental study

## Abstract

**Background:**

Current scientific evidence has pointed out the relevance of hemostatic products for improving clinical outcomes in liver trauma, including increased survival rates and reductions in bleeding-related complications. The purpose of this study was to compare the use of the gelatin-thrombin flowable (Flowable) versus the standard technique of Packing in a new experimental liver injury model.

**Methods:**

Twenty-four swine were prospectively randomized to receive either Flowable or standard packing technique. We used a novel severe liver injury model, in which the middle and left suprahepatic veins were selectively injured, causing an exsanguinating hemorrhage. The main outcome measure was the percentage of lost blood volume.

**Results:**

The median total percentage of total blood volume per animal lost, from injury to minute 120, was significantly lower in the Flowable group (15.2%; interquartile range: 10.7–46.7%) than in the Packing group (64.9%; Interquartile range: 53.4–73.0%) (Hodges-Lehmann median difference: 41.1%; 95% CI: 18.9–58.0%, *p* = 0.0034). The 24-hour survival rate was significantly higher in the Flowable group (87.0%) than in the Packing group (0.0%) (Hazard ratio (HR) 0.08; 95% confidence interval 0.102 to 0.27; *p* < 0.0001). Mean-arterial pressure was significantly lower at minute 60 and 120 in the Flowable group than in the packing group (*p* = 0.0258 and *p* = 0.0272, respectively). At minute 120, hematocrit was higher in the Flowable than in the packing group (Hodges-Lehmann median difference: 5.5%; 95%CI: 1.0 to11.0, *p* = 0.0267). Finally, the overall-surgical-procedure was significantly shorter with Flowable than with Packing (Hodges-Lehmann median difference: 39.5 s, 95% CI: 25.0 to 54.0 s, *p* = 0.0004).

**Conclusions:**

The use of the Flowable was more effective in achieving hemostasis, reducing blood loss, and improving survival rates than standard packing in a severe porcine-liver bleeding model.

**Supplementary Information:**

The online version contains supplementary material available at 10.1186/s12876-023-03114-6.

## Introduction

Hemorrhage control is one of the primary goals in the first hours of trauma surgery and remains a major potential complication [1, 2]. The development of new techniques and their early application has improved surgical outcomes [2, 3]. Since the initial description of the Pringle maneuver in 1908, additional hemostatic techniques such as perihepatic packing have been developed to facilitate hemorrhage control during liver surgery [4]. Furthermore, hemostatic agents have proven very useful for addressing this challenge of intraoperative bleeding [5]. However, selecting the right agent, at the right time, and in the right procedure, requires an adequate understanding of the mechanism of action, ideal application, and adverse effect profile of each product [6].

The use of intra-abdominal packing may tamponade life-threatening bleeding and allow optimization of organ perfusion. It may be considered as the standard procedure in severe liver trauma with massive bleeding [7, 8].

Among the available hemostatic products, the introduction of thrombin in 1970 has been an important advance for managing perioperative bleeding [6, 9]. A variety of thrombin-based hemostatic products are now available, including dressings, patches, powders, and flowable hemostats. Dressings were developed as a way to apply a dry hemostatic product for war wounds [10, 11]. Patches usually consist of a collagen fleece material coated with a dry form of fibrinogen and thrombin [12]. Fibrin sealants are two-component products, containing thrombin and fibrinogen, that mimic the final stages of the blood coagulation process. During their administration there is a rapid reaction of thrombin cleaving the fibrinogen to monomers, which leads to the formation of a fibrin meshwork [13, 14]. Finally, flowable hemostats usually combine gelatin and topical thrombin. Unlike powders, these hemostatic agents are thick and have a flowable consistency. Their main advantages include their capacity to adapt to wound geometries and fill deep lesions. Furthermore, excess material can be easily removed by irrigation [15].

Floseal™ (Baxter Healthcare Corporation Fremont, CA, USA) is a flowable hemostatic product composed of two independent hemostatic agents, namely cross-linked lyophilized bovine gelatin (500–600 μm particles) and human thrombin (500 IU/mL) [16].

Both components promote hemostasis individually. In addition, they have a synergic action, which facilitates the formation of a stable clot at the wound site. The product is biocompatible and is usually resorbed within 6 to 8 weeks [16, 17].

Researchers have reported that gelatin adheres to the liver surface by a dense fibrin network around the gelatin particles. Meanwhile, in the center of the wound, the particles of gelatin remain relatively free, with little trace of blood [17]. Additionally, thrombin plays a key role in coagulation by converting fibrin to monomers, which spontaneously polymerize and form a fibrin meshwork. Thrombin also activates many additional clotting factors, such as FVIII, FXIII and FV [18].

In the current paper, we have used liver surgery model which largely resembles an injury of liver trauma [19–21]. The novel aspect of this study was comparing the efficacy and ease of use of a gelatin-thrombin flowable hemostatic agent versus gauze packing for achieving hemostasis in a grade V liver injury, which represents a severe and bleeding injury.

This study aimed to compare the use of the gelatin-thrombin flowable (Floseal™) with the conventional standard technique of packing in a new experimental liver injury model.

## Materials and methods

### Design

A prospective, randomized, and experimental study was performed on twenty-four female swine (Large White) between 25.0 and 42.5 Kg, in the pre-clinical surgery unit of the Gómez Ulla Central Defense Hospital (Madrid, Spain). This study has been constructed, validated, published, and utilized in other studies and surgical trainings [19–21].

The study protocol (Register number: ES280790000187) was approved by the Ethics Committee, the teaching commission of the military hospital, and by the council for the environment of the community of Madrid, in accordance with Spanish and European legislation regarding animal experimentation. At the end of the study (24 h after the procedure), the animals were sacrificed with an anesthetic overdose, in accordance with current legislation.

The inclusion criterion was to have a healthy animal. Pigs were obtained from a certified supplier and quarantined by the veterinarian to prevent undisclosed illness.

Study variables were measured preoperatively (when the animal was already anesthetized), and at 12 min, 60 min, 120 min, and 24 h.

### Study groups

A review of the literature indicated that sample sizes in similar studies ranged from 3 to 12 [22, 23]. Therefore 24 pigs were randomized into two groups of 12 pigs each. The pigs were randomly selected from the herd and randomly assigned to treatment groups in a double-blinded manner prior to surgery. The surgeon was blinded to the treatment group assignments until after the injury occurred and the treatment devices were covered to prevent recognition. The same surgery team performed the experiment once weekly for two months.

Standard packing was done with 10 surgical pads measuring 20 cm × 20 cm and weighing 30 g. The gelatin-thrombin flowable hemostatic agent group (Flowable group) was done with two 5 mL syringes, containing a total of 10 mL of Floseal.

Two pigs in the Flowable group did not pass quarantine. An additional two pigs were excluded due to incomplete administration of Floseal.

### Procedures

#### Anesthetic procedure and monitoring

A venous line was selected for the infusion of drugs and fluids. Anesthesia was maintained for 120 min in all pigs using: Ketamine: 10 mgr/Kg, Midazolam: 0,5 mgr/Kg, Atropine: 0,02 mgr/Kg, Meloxicam: 0,4 mgr/Kg, Propofol: 1–1,6 mgr/Kg, Atracurio: 0,2 mgr/Kg, Fentanyl: infusion of FLK (5 vials (15 ml Fentanyl 0,05 mgr/ml + lidocaine 500 mgr + 1 ml Ketamine 100 mgr/ml), in continuous infusion at 101 ml /hour. Intubation was carried out by using a tube between 6.5 and 7.5 mm connected to a ventilator with a respiratory rate ranging between 12 and 15 breaths/min.

The animals were monitored by means of an electrocardiogram, pulse oximetry, vaginal temperature probe, and capnography. In addition, a femoral arterial probe was used for invasive monitoring of blood pressure and heart rate.

We evaluated the following analytical determinations: prothrombin time, partial thromboplastin time, pH, PCO_2_, PO_2_, Base excess (BEB), HCO_3_^−^, Na^+^, K^+,^ Ca^++^, Glucose, hematocrit, and hemoglobin.

The amount of fluid administered during the procedure was equivalent to the volume of blood lost.

Animals did not recover from anesthesia during the 24 h observation period.

### Surgical procedure

An extended right subcostal laparotomy was performed. Afterwards, the middle (segment IV) and left (segments II and III) suprahepatic veins were located by echo-Doppler (Logic V2, General Electric, Chicago IL, USA).

To standardize the injuries and avoid bias, two identical incisions were made on the liver parenchyma for each case. Each incision was 2 cm long and 5 cm deep, created using a No. 20 scalpel blade. Liver injuries were also standardized by using ultrasonography for localization during the procedures. Post-mortem analysis was then used to confirm complete transection of vessels in all cases.

**Fig. 1:**
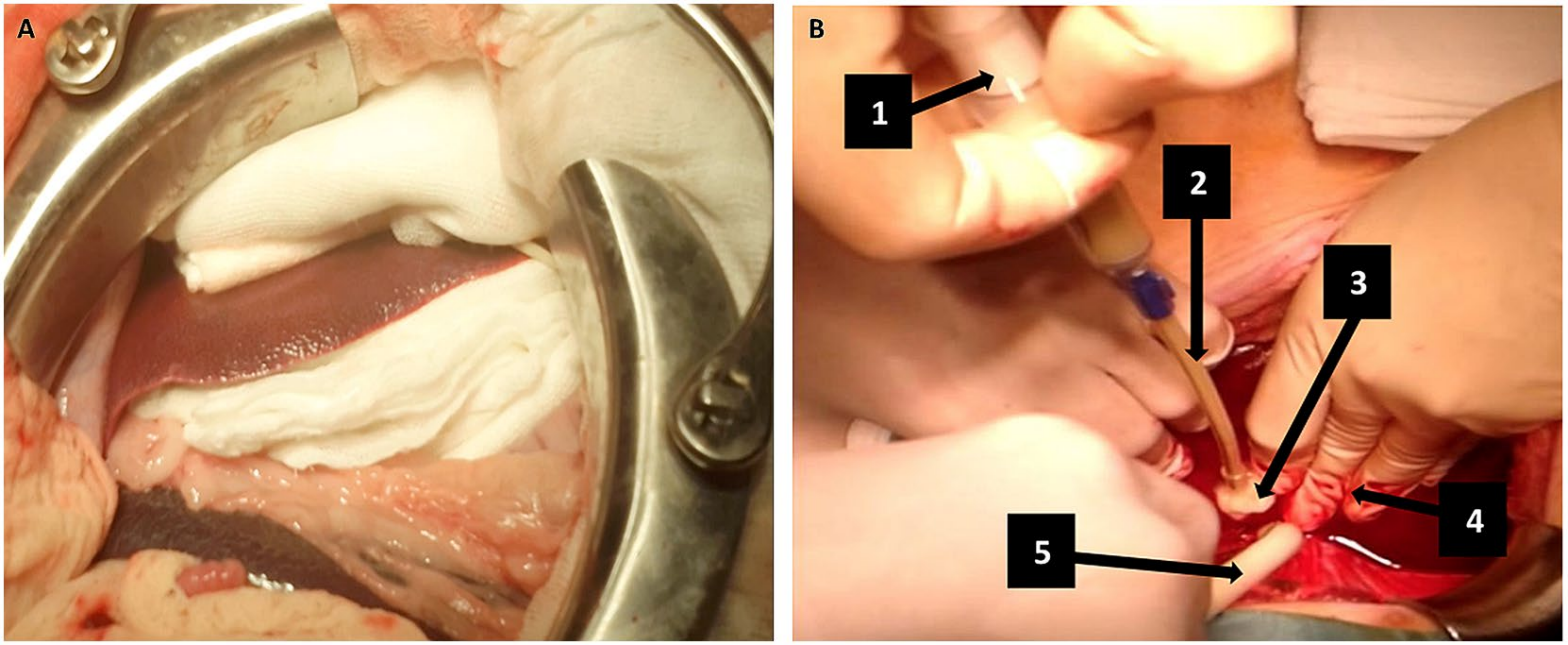
Hemostatic techniques used in the study. (**A**). Liver packing application technique (Fig. 1A). A total of 10 compresses were placed around the liver, applying an even pressure. (**B**). Technique of Floseal™ application on the liver injury. (Fig. 1B). (1) Two syringes of 5 mL with Floseal™ are applied during the first three minutes. (2) Applicator attached to the syringe. (3) Floseal™ was administered into in the wound. (4) Direct compression with gauze by hand. (5) Vacuum cleaner

After injury, the control group underwent the standard packing technique with sterile surgical pads (Fig. 1). After 12 min, the packing was removed, hemostasis was checked, and the packing was subsequently repeated. We removed all ten gauzes, from the outer layers to those that were in direct contact with the liver surface, in order to quantify the blood loss. Two additional controls, at 60 and 120 min, were carried out. During this time the packing was maintained.

**Fig. 2:**
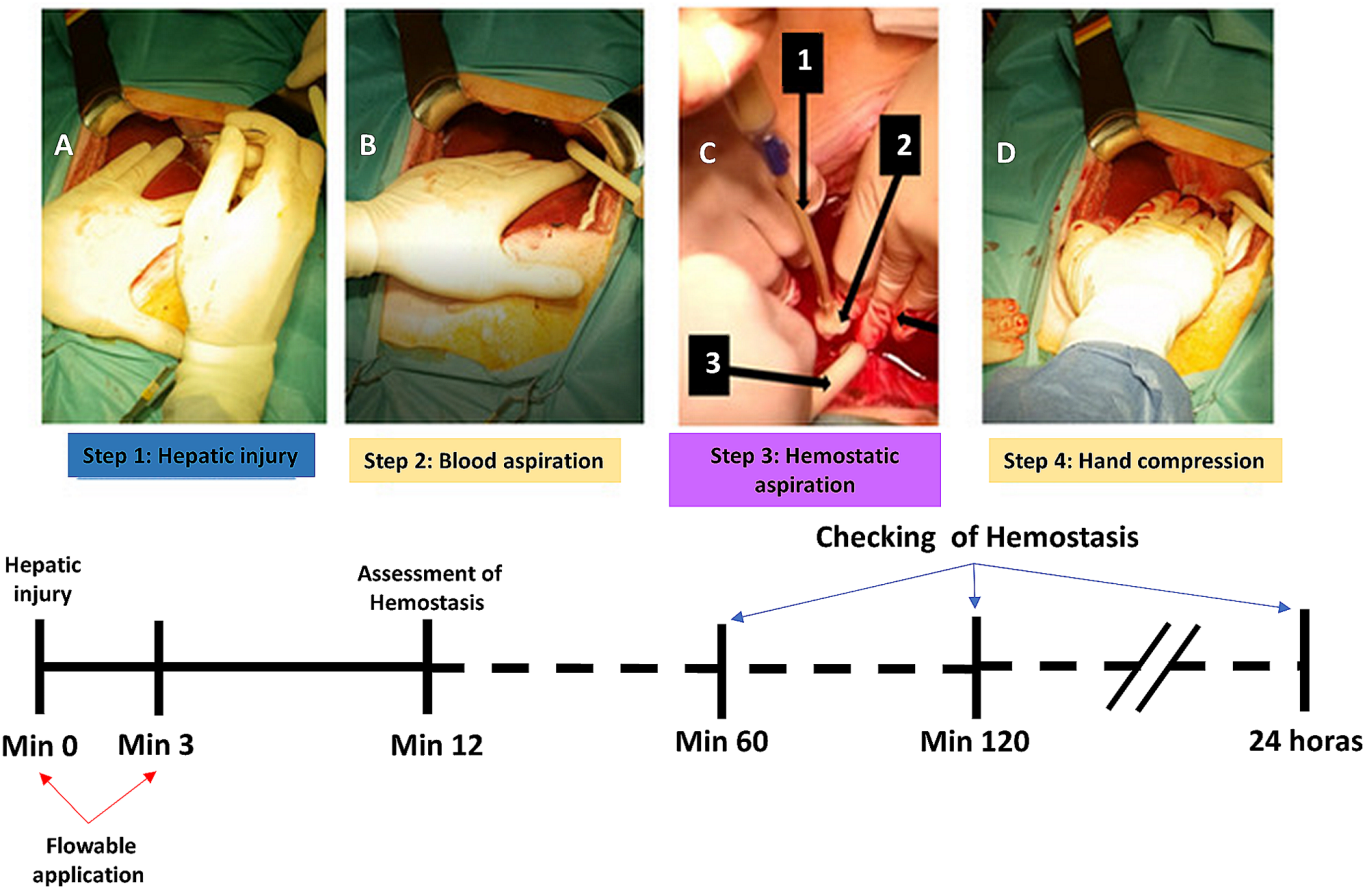
Timeline of the study groups. (**A**) Liver injury. (**B**) Assessment of blood loss. (**C**) Floseal application. 1: Applicator attached to the syringe; 2: Administration of Floseal into the wound; 3: Aspiration of blood lost. There was a 3-minute window (see red arrows) for applying additional Floseal. (**D**) Hand compression with gauze

Floseal™ was prepared from the Flowable group and applied according to its “Instructions for Use” (Fig. 2, steps 3–4). Two 5 mL syringes were initially applied over the first three minutes. As summarized in Fig. 2, the procedure timeline involved applying Floseal™ followed by 12 min of hand compression with gauze. Hemostasis was then checked after removing the compression. The 12-minute compression time was selected based on our pilot study findings that injuries typically required at least 12 min to achieve initial hemostatic control. To balance monitoring hemostasis at regular intervals without excessively disturbing the injury site, we divided the 12-minute period into three-minute intervals for assessment.

This maneuver equals the needed compression time for both groups in the first minutes after injury. Afterward, no further compression was provided to the hemostatic group in order to allow the hemostatic agent to work without any additional bias from compression methods.

Hemostasis was evaluated again at 60 and 120 min, and at 24 h.

### Primary outcomes

The primary outcome measures were the percentage of total blood volume per animal lost.

The blood was removed using a surgical aspirator (Flexivac®) and gauze packing pads. The volume of the blood loss was calculated with this formula: “v = [(b1-a1)+(b2-a2)]/1.04”, where “b1” is the weight of the tank of the surgical aspirator loaded with blood, “a1” the dry weight of the tank (without blood), “b2” the weight of the surgical pads soaked in blood, and “a2” the dry weight of surgical pads (without blood) and 1.04 is the constant of pig´s blood density.

The total blood volume was estimated to be 66 mL/kg [24]. Volume of blood loss was finally expressed as a percentage of total blood volume, according to the formula V% = Vx100/Ve, where “V%” was the percentage of blood lost, “V “was the volume of blood lost (mL), and “Ve” was the estimated volume of blood (mL) according to the weight of the pig.

The percentage of total blood volume per animal lost was measured in both groups from 0 to minute 12; from minute 12 to minute 60; from minute 60 to minute 120, and from 0 to hour 24.

The load aspirator and the soaked gauzes was weighted in a weighing scale (Mini SP; Mobba; Barcelona, Spain) model.

Balance calibration, measuring testing, and validation were done according to the supplier instructions. Repeated measurements were replicable and reproducible in previous studies published by our group [19].

#### Secondary outcomes

The secondary outcomes measured were survival rate; time of application; heart rate (beats per minute, bpm); mean arterial pressure; hemoglobin; and hematocrit values.

The amount of time needed to apply the hemostat was used as an indicator of the difficulty of applying the hemostatic agent. It was assumed that a longer time of application correlated with a greater degree of difficulty.

### Statistical analysis

A standard statistical analysis was performed using MedCalc Statistical Software version 20.104 (MedCalc Software bv, Ostend, Belgium; https://www.medcalc.org; 2022).

Data were tested for normal distribution using a D’Agostino-Pearson test.

Descriptive statistics number (percentage); mean (standard deviation, SD); median (interquartile range, IqR); or median (95% confidence interval, CI) were used, as appropriate.

The comparisons of the blood loss and hemodynamic parameters were performed using a Friedman’s two-way analysis test.

The Mann-Whitney U test was used for comparing different parameters between the Flowable and the Packing groups.

Survival rates were plotted for study groups using a Kaplan–Meier analysis and were compared using a log-rank test.

Linear regression analysis was used to assess the relationship between time of application, as an independent variable, and the volume of blood lost at minute 12, as a dependent variable.

Categorical variables were compared using a Chi-square test and a Fisher’s exact test, as needed.

A P-value of less than 0.05 was considered significant.

## Results

### Preoperative values

Twenty female swine, 12 pigs in the control group and 8 pigs in the Flowable group, were included in the study. All animals had a body weight ranging from 25 to 42 kg.

Table 1 summarizes main baseline clinical characteristics.

**Table 1:** A comparison of the main baseline clinical between standard packing (control group) and FloSeal™ hemostatic agent (sealant group)

Variable	Sealant	Control
Weight, kg		
Mean (SD)	33.6 (6.3)	34.2 (2.4)
Ve*, mL		
Mean (SD)	2047.3 (381.8)	2084.2 (143.8)
Temperature, °C		
Mean (SD)	34.8 (0.9)	35.4 (1.3)
Heart rate, bpm		
Mean (SD)	87.0 (21.2)	80.6 (9.3)
SBP, mm Hg		
Mean (SD)	91.6 (13.4)	90.1 (16.4)
DBP, mm Hg		
Mean (SD)	54.1 (9.7)	55.6 (12.0)
MAP**, mm Hg		
Mean (SD)	69.9 (11.7)	66.9 (15.3)
Hemoglobin, g/dL		
Mean (SD)	9.4 (1.5)	8.9 (1.4)
Hematocrit, %		
Mean (SD)	26.8 (4.1)	24.8 (6.6)
PT, sec		
Mean (SD)	14.0 (2.7)	14.7 (4.4)
PTT, sec		
Mean (SD)	14.3 (4.0)	19.2 (7.5)
Ringer Lactato, mL		
Mean (SD)	268.0 (66.4)	332.7 (62.5)
Glucose, mg/dL		
Mean (SD)	61.4 (10.4)	66.8 (12.9)
Sodium, mEq/L		
Mean (SD)	138.8 (1.2)	139.0 (1.8)
Potassium, mEq/L		
Mean (SD)	3.9 (0.6)	3.6 (0.3)
Calcium, mEq/L		
Mean (SD)	1.36 (0.04)	1.33 (0.07)
pH		
Mean (SD)	7.49 (0.04)	7.49 (0.04)
Excess base, mmol/L		
Mean (SD)	9.1 (2.0)	8.0 (2.6)
Bicarbonate, mEq/L		
Mean (SD)	33.2 (1.8)	31.9 (2.6)
PCO_2_, mm Hg		
Mean (SD)	42.6 (3.0)	41.4 (4.0)
TCO_2_, mm Hg		
Mean (SD)	34.5 (1.9)	33.2 (2.7)
PO_2_, mm Hg		
Mean (SD)	184.1 (58.7)	210.4 (61.4)
SO_2_, %		
Mean (SD)	99.5 (0.9)	100.0 (0.0)
AO_2_S, %		
Mean (SD)	99.1 (1.5)	99.4 (1.5)

SD: Standard deviation; Ve: Estimated total blood volume; bpm: Beats per minute; SBP: Systolic blood pressure; DBP: Diastolic blood pressure; MAP: Mean arterial pressure; PT: Prothrombin time; PTT: Partial thromboplastin time; BSS: Balanced saline solution; PCO_2_: Partial pressure of carbon dioxide; TCO_2_: Total CO_2_ in blood; PO_2_: Partial pressure of oxygen; O_2_S: Oxygen saturation; AO^2^S: Arterial Oxygen Saturation

*Estimated Blood volume = Weight of the animal × 61mL/kg (see reference 24)

**Calculated according to the formula: DBP + 1/3 (SBP-DBP)

With the exception of the amount of balanced saline solution administered, which was significantly greater in the control group (*p* = 0.0278), there were no significant differences in any of the clinical or analytical parameters between the control and the Flowable groups (Table 1).

### Primary endpoints

In the Flowable group, the median (IqR) percentage of total blood volume per animal lost was 14.6% (8.6–37.8%) from injury to minute 12; 1.1% (0.2–4.2%) from minute 12 to minute 60; 0.3% (0.0 to 0.4%) from minute 60 to minute 120; and 15.2% (9.9–43.2%) from injury to minute 120; *p* < 0.0001, Friedman rank sum test.

In the standard packing group, the median (IqR) of blood volume lost was 31.3% (23.4–35.8%) from injury to minute 12; 21.2% (15.7–24.2%) from minute 12 to minute 60; 10.3% (0.0–17.0%) from minute 60 to minute 120; and 64.9% (53.4–73.0%) from injury to minute 120; *p* < 0.0001, Friedman rank sum test.

**Fig. 3:**
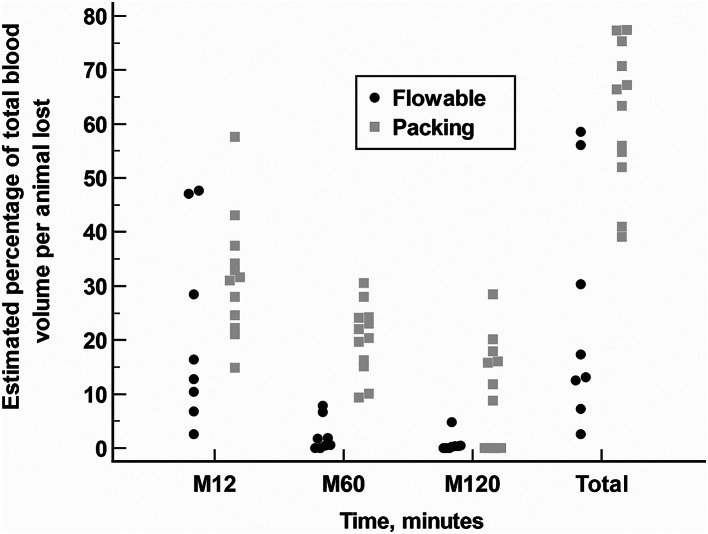
Dot plot analysis comparing the estimated blood volume lost (%) between the standard packing and the Floseal™ hemostatic agent (Flowable). The estimated percentage of total blood volume per animal lost (VBL) was calculated according to the formula: VBL= (V/Ve) × 100. “V” was the volume of blood lost (mL), and “Ve” was the estimated volume of blood (mL) according to the weight of the pig. Statistical significance was calculated by Mann-Whitney U test. M 12: Hodges-Lehmann median difference: 13.3%; 95% CI: -6.2–24.6%, *p* = 0.1228. M 60: Hodges-Lehmann median difference: 18.9%; 95% CI: 13.1–23.1%, *p* = 0.0002. M120: Hodges-Lehmann median difference:8.8%; 95% CI: 0.3–16.1%, *p* = 0.0431. Total: Hodges-Lehmann median difference: 41.1%; 95% CI: 18.9–58.0%, *p* = 0.0034. M12: VBL from injury to minute 12; M60: VBL from minute 12 to minute 60; M120: VBL from minute 60 to minute 120; Total: Total of VBL from injury to minute 120

With the exception of minute 12 measurements (Hodges-Lehmann median difference: 13.3%; 95% CI: -6.2–24.6%, *p* = 0.1228), The percentage of lost blood volume per animal was significantly lower in the Flowable group than in the packing group from minute 12 to minute 60 (Hodges-Lehmann median difference: 18.9%; 95% CI: 13.1–23.1%, *p* = 0.0002); from minute 60 to minute 120 (Hodges-Lehmann median difference: 8.8%; 95% CI: 0.3–16.1%, *p* = 0.0431); and from injury to minute 120 (Hodges-Lehmann median difference: 41.1%; 95% CI: 18.9–58.0%, *p* = 0.0034) (Fig. 3).

The total volume of blood loss, from injury to minute 120, was significantly lower in the Flowable group than in the packing group (Hodges-Lehmann median difference: -952.5 cc; 95% CI: -1,323.0 to -435.0 cc; *p* = 0.0020).

### Secondary outcomes

**Fig. 4:**
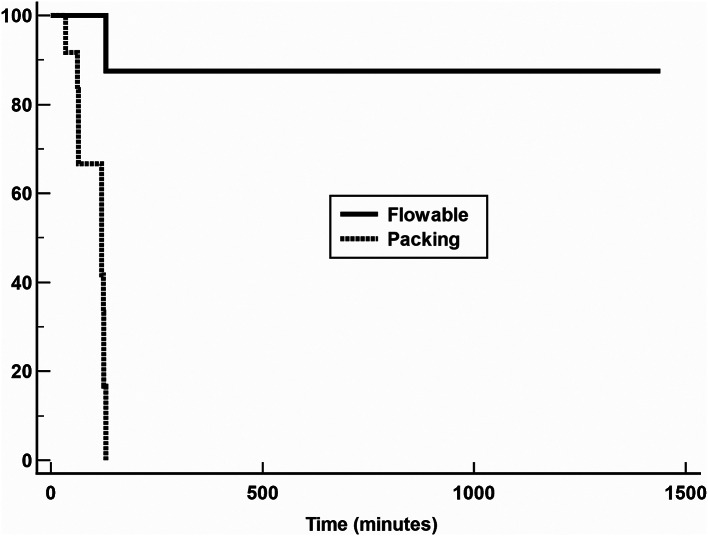
Kaplan–Meier survival curves for failure in Flowable (solid line) and packing (dotted line) groups. Death occurred in 1 (12.5%) Flowable-treated pigs and in 12 (100.0%) packing-treated pigs. Hazard ratio (HR) 0.08, 95% confidence interval 0.102 to 0.27); *p* < 0.0001

Kaplan–Meier survival analysis indicated a significantly lower risk for death in the animals treated with the Flowable (Hazard ratio: 0.08; 95% CI: 0.102 to 0.27; *p* < 0.0001) (Fig. 4).

**Fig. 5:**
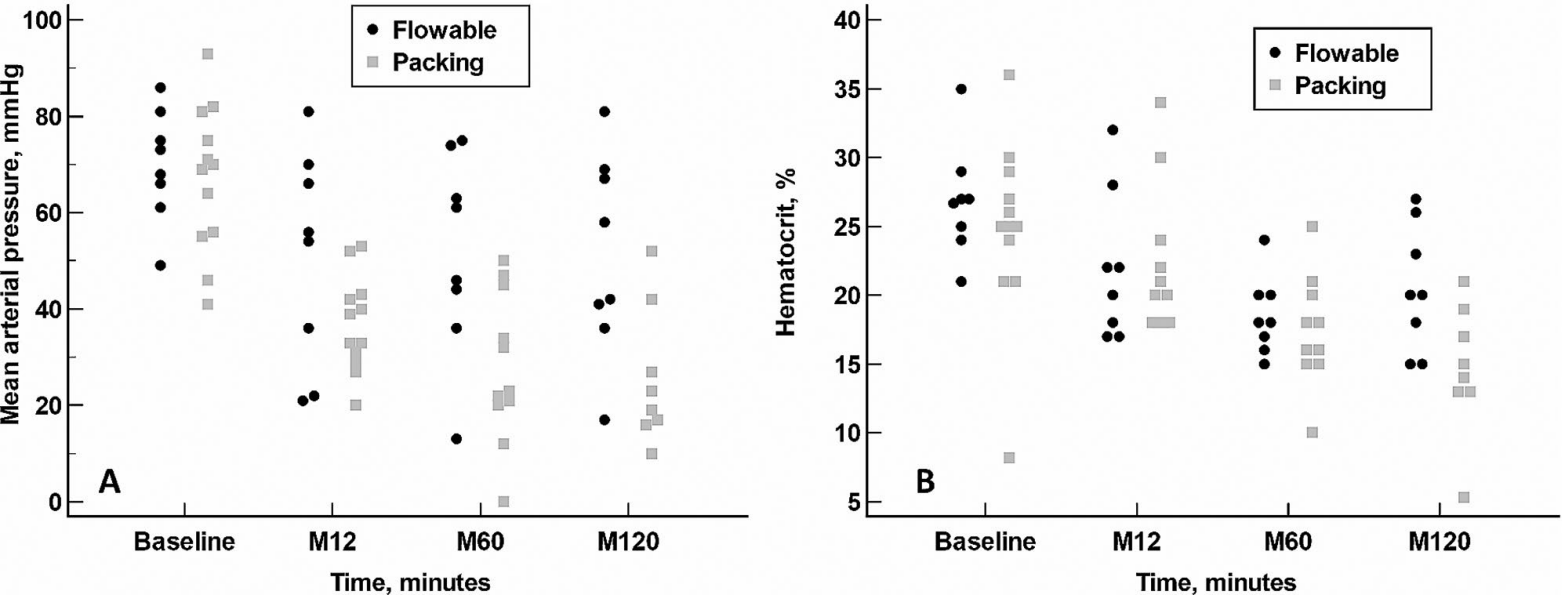
Dot plot analysis comparing mean arterial pressure (5 A) and hematocrit (5B) parameters throughout the study in the Flowable and packing groups. Minute 60: Hodges-Lehmann median difference: 24.5 mm Hg; 95% CI: 2.0 mm Hg to 43.0 mm Hg, *p* = 0.0258. Minute 120: Hodges-Lehmann median difference: 25.0 mm Hg; 95% CI: 6.0 mm Hg to 48.0 mm Hg, *p* = 0.0272. Statistical significance was calculated by Mann-Whitney U test (differences between groups)

In the Flowable group, blood pressure remained stable throughout the study (Friedman test, *p* = 0.9675). However, in the packing group, there was significant blood pressure instability (Friedman test, *p* = 0.0002) (Fig. 5A).

As compared to baseline, hematocrit was significantly lower at all time-points evaluated. Hematocrit gradually recovered over the course of the evaluation, although values had not fully recovered at minute 120 (Fig. 5B). At minute 120, hematocrit was significantly higher in the Flowable group (median: 20.0%; 95% CI: 15.0–26.2%) than in the packing group (median: 14.5%; 95% CI: 11.5–19.4%) (Hodges-Lehmann median difference: 5.5%; 95% CI: 1.0–11.0%, *p* = 0.0267).

**Fig. 6:**
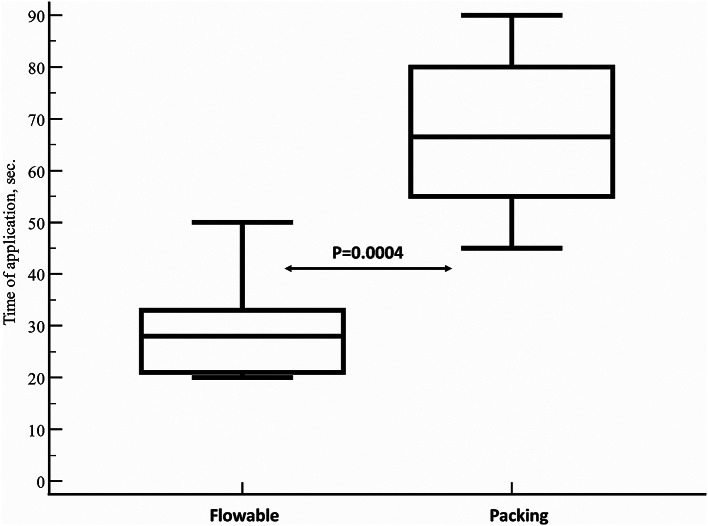
A comparison of the time of application between Flowable and packing groups. Vertical bars represent 95% confidence interval. The time of application was significantly lower in the Flowable (median: 28.0 s; 95% CI: 20.8 to 36.2 s) group than in the packing group (median: 66.5 s; 95% CI: 51.7 to 80.0 s) (Hodges-Lehmann median difference: 39.5 s, 95% CI: 25.0 to 54.0 s, *p* = 0.0004). Statistical significance was assessed by Mann-Whitney U test

The time spent on administration was significantly lower in the Flowable group (median: 28.0 s; 95% CI: 20.8 to 36.2 s) than in the packing group (median: 66.5 s; 95% CI: 51.7 to 80.0 s) (Hodges-Lehmann median difference: 39.5 s, 95% CI: 25.0 to 54.0 s, *p* = 0.0004) (Fig. 6).

**Fig. 7:**
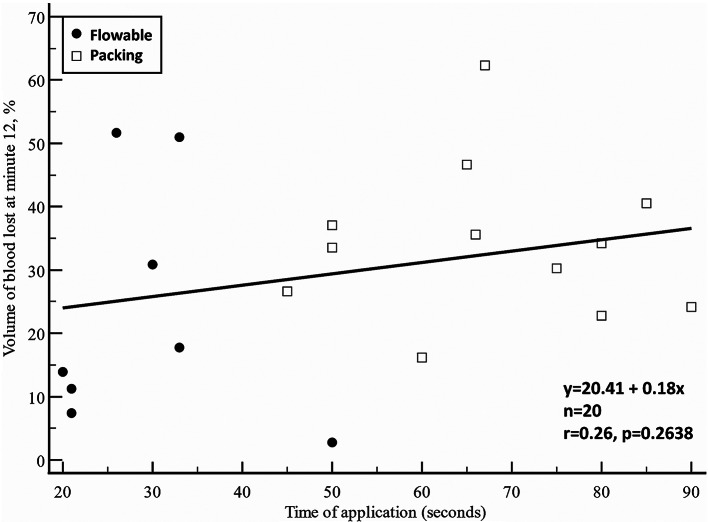
Scatter plot showing the relationship between the time of application and the volume of blood lost at minute 12. Flowable group: *r* = 0.08, *p* = 0.8431, regression line slope − 0.16%/sec; where 95% CI ranged from − 2.08 to 1.76%/sec. Packing group: *r* = 0.03, *p* = 0.8431, regression line slope − 0.03%/sec; where 95% CI ranged from − 0.61 to 0.56%/sec. Comparison between slopes: mean difference: -0.13, standard error: 0.70, *p* = 0.8503

Linear regression analysis did not show a significant correlation between the time needed for application and the volume of blood lost at minute 12 in either the Flowable group (*r* = 0.08, *p* = 0.8431, regression line slope − 0.16%/sec; where 95% CI ranged from − 2.08 to 1.76%/sec) or in the packing group (*r* = 0.03, *p* = 0.8431, regression line slope − 0.03%/sec; where 95% CI ranged from − 0.61 to 0.56%/sec). Overall, there were no significant differences in slopes between the two groups (mean difference: -0.13, standard error: 0.70, *p* = 0.8503) (Fig. 7).

Linear regression analysis did not show a significant correlation between the time needed for application and the volume of blood lost at minute 12 in either the Flowable group (*r* = 0.08, *p* = 0.8431, regression line slope − 0.16%/sec; where 95% CI ranged from − 2.08 to 1.76%/sec) or in the packing group (*r* = 0.03, *p* = 0.8431, regression line slope − 0.03%/sec; where 95% CI ranged from − 0.61 to 0.56%/sec). Overall, there were no significant differences in slopes between the two groups (mean difference: -0.13, standard error: 0.70, *p* = 0.8503) (Fig. 7).

## Discussion

The results of our study suggested that the Flowable product provided better hemostatic control than standard packing technique in a novel experimental liver injury model. Additionally, the survival rate was significantly higher in the Flowable group.

According to the Floseal™ data sheet, the product should be topically applied to a bleeding site, as an adjunct to the hemostasis, when traditional methods for controlling bleeding are ineffective or impractical [16]. FloSeal™ has been successfully used in different types of surgeries [25–30].

Although this evidence is extremely useful in daily practice, there is a continuing need to develop experimental studies to provide high-quality evidence in different clinical settings.

### Thrombin hemostatic agents versus packing

As far as we know, this is the first study comparing the use of standard packing with the gelatin-thrombin flowable Floseal™ in an experimental model of severe liver injury.

It has been previously suggested that volume of blood loss was lower when using a hemostatic agent than when using the standard packing technique [5, 31–33]. In agreement with their results, we found that at minute 12, the volume of blood lost was 13.3% lower in the Flowable group. Additionally, the use of the Flowable significantly reduced the risk of death.

In the Flowable group, blood pressure data showed no significant changes throughout the first 120 min after the injury, which highlights the hemodynamic stability obtained in that group with the use of the flowable hemostatic agent. On the contrary, in the packing group, there were significant changes in heart rate suggestive of hemodynamic instability.

As compared to baseline, hematocrit values were significantly lower in both groups at all time points measured. Nevertheless, hematocrit gradually recovered as time passed in the Flowable group, but remained low in the packing group.

Since weight may significantly influence blood volume [25], we used the percentage of total blood volume per animal lost ([volume of blood loss/total blood volume] ×100) as the primary endpoint.

The lack of statistical significance between the Flowable group and packing at minute 12 may be explained by the study methodology. The first determination of the percentage of total blood volume per animal lost in the packing group was from 0 to minute 12.

### Gelatin-thrombin flowable agents

The hemostatic effectiveness of the gelatin-thrombin flowable product was evaluated in a severe traumatic liver and spleen rupture model in swine [34].

Regarding survival, our study found a survival rate of 100% at minute 120 and 87% after 24 h in the Flowable group, which is in line with the results of Leixnering et al., who reported a survival rate of 100% in the FlosealT^M^ group [34].

Although we did not measure the time required to reach hemostasis, it should be noteworthy that the amount of blood lost at minute 12 gradually decreased from minutes 12 to 60 and from minutes 60 to 120 (Friedman test, *p* = 0.0492).

### Ease of use

Previously, there have not been studies which evaluated the time needed to apply the gelatin-thrombin matrix or its impact on outcomes. The time taken to apply the flowable in our study was significantly shorter than that seen in the packing group.

We did not find any relationship between the time taken to apply the hemostat and the percentage of total blood volume per animal lost at minute 12.

The current study has some limitations that should be taken into consideration when interpreting its results. The first one is the fact that the study was conducted on a novel experimental animal model. Therefore, we must be cautious when applying these findings in clinical practice. The second one was its open label design; further studies are needed to provide stronger evidence of durable results.

## Conclusions

The results of the current study clearly showed that the gelatin-thrombin flowable FloSeal™ provided a better hemostatic profile than standard packing in a novel experimental liver injury model in pigs. Additionally, the use of the gelatin-thrombin flowable resulted in significantly improved survival rates. Moreover, according to surgeon experience, the gelatin-thrombin flowable was easy to use and provided good hemostasis, noting that the current study used the time taken to apply the hemostat as an indicator of the difficulty of applying the hemostat.

### Electronic supplementary material

Below is the link to the electronic supplementary material.

**Supplementary Material 1: Figure S1.** Dot plot analysis comparing heart rate (S1A) and hemoglobin (S1B) parameters throughout the study in the Flowable and packing groups. bpm: beats per minute

## Data Availability

Data and material are available on request due to ethical or legal reasons at franciscosanchez@healthgood.es.

## Declarations

All methods are reported in accordance with ARRIVE guidelines for the reporting of animal experiments.
