# Supplementary material for: Effectiveness of a thrombin-gelatin flowable for treating severe liver bleeding: an experimental study
Source: BMC Gastroenterol. 2024 Feb 14;24:71. doi: 10.1186/s12876-023-03114-6 (PMC10865537; doi:10.1186/s12876-023-03114-6)

**Supplementary Material**

Figure S1. Dot plot analysis comparing heart rate (S1A) and hemoglobin (S1B) parameters throughout the study in the Flowable and packing groups.

bpm: beats per minute.


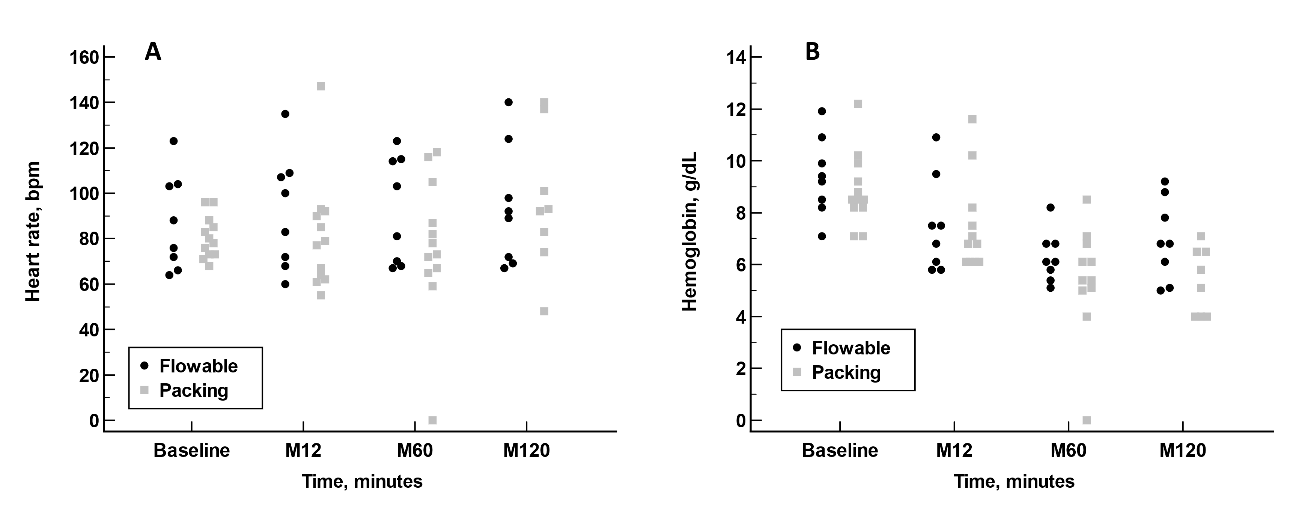

Supplement: Supplementary file 1 — Supplementary Material 1: Figure S1. Dot plot analysis comparing heart rate (S1A) and hemoglobin (S1B) parameters throughout the study in the Flowable and packing groups. bpm: beats per minute [file 12876_2023_3114_MOESM1_ESM.docx]
